# Moral Foundations Questionnaire and Moral Foundations Sacredness Scale: Assessing the Factorial Structure of the Dutch Translations

**DOI:** 10.5334/pb.1188

**Published:** 2023-07-24

**Authors:** Ann De Buck, Lieven J. R. Pauwels

**Affiliations:** 1 Ghent University, Belgium

**Keywords:** moral foundations questionnaire, moral foundations sacredness scale – Dutch translation, measurement invariance, factor structure

## Abstract

The Moral Foundations Questionnaire (MFQ) and the Moral Foundations Sacredness Scale (MFSS) have been proposed to advance conceptualizations of morality. This study assesses the factor structure of the Dutch translations of the short version of the MFQ (20 items) and the full MFSS. The five-factor model posited by Moral Foundations Theory (MFT) is compared against alternative models of morality. Correlational analyses are performed between the best-fitting models. A multi-group confirmatory factor analysis of the optimal model is tested across gender. Data are taken from an online survey of a student sample (*N* = 1496). Results suggest that the Dutch translation of the MFQ20 does not converge on the proposed five-factor model. Conversely, MFSS subscales show good model fit, but intercorrelations among the five subscales are high. Weak invariance is retained for MFSS but not for MFQ20. Overall, the present study shows that the Dutch version of the MFSS scale performs better than the MFQ20 in terms of scale reliability, fit indices, and measurement invariance testing. More methodological inquiries on MFSS are welcomed, whereas the use of the MFQ20 should be discouraged. Instead, researchers on moral foundations are encouraged to empirically test the psychometric properties of the recently revised MFQ-2, developed by the authors of MFT as a more accurate instrument for the conceptualization of morality.

## Introduction

The Moral Foundations Theory (MFT) (Graham, Nosek, Haidt, Iyer, Koleva & Ditto, 2011; Haidt & Graham, 2007; Haidt & Joseph, 2004) is a contemporary cultural-psychological account of moral judgment and decision-making. The origins of MFT can be traced from a review of the literature in evolutionary psychology and anthropology on morality across cultures (Graham, Haidt, Koleva, *et al*., 2012). MFT proposes a small set of innate and universally available psychological systems upon which each culture constructs unique moralities (moralfoundations.org). Because humans face multiple social problems, they rely on multiple moral intuitions – “foundations” – when making moral decisions (Haidt, 2013). The five foundations of morality are hypothesized as Care: the tendency to prevent any kind of harm to others, Fairness: the tendency to avoid unfair treatment, and cheating and to uphold abstract notions of justice and rights, Loyalty: the tendency to fulfill obligations of group membership including self-sacrifice, Authority: the tendency to respect and maintain the social order, traditions of society and obligations of hierarchical relationships such as obedience, and Sanctity: the tendency to avoid physical, spiritual contamination, linked to the emotion of disgust. The conceptualization of MFT comes in the form of two quantitative self-report measures: the Moral Foundations Questionnaire (MFQ) and the Moral Foundations Sacredness Scale (MFSS). Both questionnaires are developed to provide reliable, valid, and distinct measurement instruments of the moral domain, grounded in theory, that are broader than empathy and justice concerns assessed by existing measures of moral concerns (Graham *et al.,* 2011). The scales are translated into a wide range of languages (available on moralfoundations.org) that allow testing the cross-cultural validity of MFT’s claim of the multi-foundational conception of morality. The present study assesses the factor structure of the Dutch translations of the short version of the MFQ (20 items) and the full MFSS (20 items) among a large sample of university students (*N* = 1496).

## Overview of the Study

**Figure 1:**
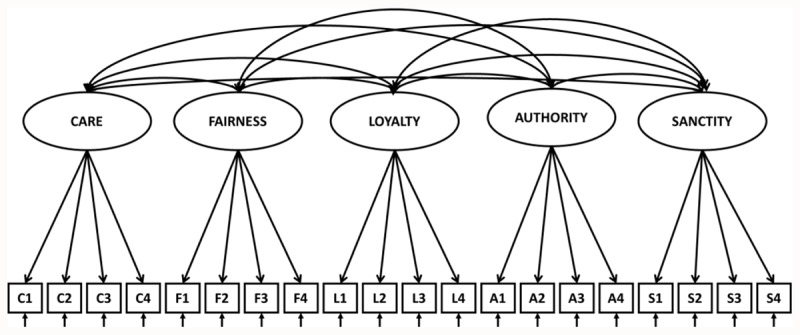
Hypothesized best fitting model 1: Five correlated factors model.

Our main objective is to test the five-factor structure of the 20 items short version of the Moral Foundations Questionnaire (henceforth MFQ20) and the Moral Foundations Sacredness Scale (henceforth MFSS) against several competing theoretical factor structures. The confirmatory factor analyses (CFA) test procedures draw upon previous validation studies by Graham, Haidt, and Nosek (2009) and Graham *et al*. (2011), although the latter did not include a CFA test of the MFSS. The present study has three goals. The first goal is a comparison of nested first-order factor structures. Several measurement models are created that compare different theoretically derived factor structures. The hypothesis is that a five correlated factors model: Care, Fairness, Loyalty, Authority, and Sanctity (Figure 1) provides a better overall model fit than a single morality factor model (Appendix 4), a two-factor model corresponding to Individualizing and Binding foundations (Appendix 5), and a three-factor model corresponding to Shweder’s ethics of Autonomy, Community, and Divinity (Shweder, Much, Mahapatra, & Park 1997) (Appendix 6). CFA analyses are conducted on the MFQ20 items scale and on the MFSS (20 items) scale. Our second goal is to compare correlations among and between MFQ20 and MFSS subscales. It is hypothesized that higher correlations will be found between respectively Care and Fairness as measures of Individualizing foundations and Loyalty, Authority, and Sanctity as measures of Binding foundations. Our third goal is to test factorial invariance in the hypothesized five-factor CFA model across gender.

## Previous validation studies

The development and validity tests of MFQ are extensively described in a key study by Graham *et al*. (2011), in which the five moral foundations structure is proposed as a reliable, valid, and easy-to-use measurement tool for exploring the moral domain (p. 382) To advance future validation studies in other populations, MFQ has been translated into a wide range of languages (available at MoralFoundations.org) and utilized to test MFT’s five-foundational propositions in other cultures and countries. Support for a five-factor structure is found in three Chinese ethnic groups (Du, 2019), Brasil (Moreira, de Souza & Guerra, 2019), New Zealand (Davies, Sibley & Liu, 2014), Sweden (Nilsson & Erlandsson, 2015); Turkey (Yalçindag *et al.*, 2019; Yilmaz *et al.,* 2016), although it is acknowledged that five-factor models provide poor fit to the data (Davis, Rice, Van Tongeren *et al.,* 2016; Zakharin & Bates, 2021). In addition, there is some evidence that the five-factor model proposed by MFT is not generalizable and, as a result, may not be meaningfully compared across populations. For instance, Iurino and Saucier (2020) were unable to replicate MFT’s five-factor structure using MFQ20 across 27 countries suggesting that the proposed five-factor model is not cross-culturally valid. By contrast, Doğruyol, Alper, and Yilmaz (2019) provide evidence for a stable five-factor model, operationalized by the short version of the MFQ, across WEIRD and non-WEIRD^1^ cultures, although Atari and colleagues (Atari, Haidt, Graham *et al.,* 2012) have questioned the problematic dichotomy of WEIRD vs. non-WEIRD societies. In contrast, only a few studies assessed the construct validity of the scales in MFSS. For instance, acceptable fit indexes for the proposed five correlated factor model of the MFSS were found in a male Spanish sample (Vecina, 2014), three samples in Turkey (Yalçındağ *et al*. 2019), and a large sample of volunteers at www.yourmorals.org, mainly from the United States (82.12%) (Graham, Haidt & Nosek, 2009).

## Method

### Participants

Data were collected from undergraduates at a large university in Ghent, a city in the northern region of Belgium. Students did not receive course credit for their participation. Students participated in an online survey that incorporated the Dutch translations of the MFQ20 and the MFSS. The sample is a convenience sample of 1496 students (mean age = 19.89, *SD* = 3.20; 29.1% men), studying a wide range of sciences (sample descriptives in Appendix 1).

### Measures

The Dutch version of the MFQ20 has been translated into Dutch by van Leeuwen and back-translated to English by Spiering (MoralFoundations.org). MFSS was independently translated into Dutch by the authors. MFQ (Appendix 2) is a self-report measure of the degree to which individuals endorse each of five intuitive moral concerns posited by MFT: Care, Fairness, Loyalty, Authority, and Sanctity. MFQ is originally made up of 30 items but also exists in a 20-item short form (MFQ20) that is the scale under study. MFQ20 is split into two subscales: *Relevance* and *Judgment*. The subscale *Relevance* measures abstract self-assessments of what elements are of moral relevance to foundation-related considerations. The second subscale *Judgement* measures agreement with contextualized specific moral statements supporting or rejecting foundations-related concerns (Graham *et al.,* 2011). Both subscales include ten items, two for each foundation, categorically scored on a five-point scale (1 = completely disagree/not at all relevant to 5 = completely agree/extremely relevant). MFSS (Appendix 3) is a self-report measure, developed by Graham, Haidt, and Nosek (2009). Participants are presented with potential violations of the five moral foundations (e.g. “Kick a dog in the head, hard” mapping onto the Care foundation) and asked how much money they would require to do it. The idea is that different moral foundations may be sacred for some people but not for others. By sacred, it is meant that participants would not for any amount of money violate the principles of that foundation (YourMorals.org). The scale gives four items for each foundation, responded to on a seven-point scale, from “€0 (I’d do it for free),” then €10, and then increasing by factors of 10 to a million euros, with a top option of “never for any amount of money” (Graham & Haidt, 2012).

### Analytic strategy

Confirmatory factor analyses (CFA) were performed in M*plus* version 7.11 (Muthén & Muthén, 2012). Weighted Least Squares Means and Variances (WLSMV) were utilized for model estimation (Kline, 2016). Subsequently, measurement invariance (MI) was tested using multi-group first-order CFA for categorical variables (Bollen, 1989). At each step of the MI procedure, a series of nested factor models, that place increasing restrictions on parameters across the groups, were estimated (Widaman, 1985). When WLSMV is used for model estimation, the DIFFTESToption in M*plus* is available for difference testing (Muthén & Muthén, 2012). Model fit was assessed using the following indices: A non-significant χ^2^ is desired. However, χ^2^ statistic is highly sensitive to sample size. As such, the significance of the χ^2^ test should not be a reason by itself to reject a model (Wang & Wang, 2020); Comparative fit index (CFI) (Bentler, 1990) and Tucker Lewis Index (TLI; Tucker & Lewis, 1973) (>0.95 (Hu & Bentler, 1999)); Root mean square error of approximation (RMSEA), interpreted as 0 = perfect fit; <0.05 = close fit; 0.05–0.08 = fair fit; 0.08-.0.10 = mediocre fit; and >.10 = poor fit (Byrne, 2012; Hu & Bentler, 1999; MacCallum *et al.,* 1996). In addition, the 90% CI, computed for the RMSEA is reported. Ideally, the lower value of the 90% CI should be very near zero (or no worse than 0.05) and the upper value should be less than 0.08 (Steiger, 2007); weighted mean-square residual standardized (WRMR), a residual-based model fit index. Perfect model fit is indicated by WRMR = 0 and increasingly higher values indicate a worse fit (Kline, 2016).

## Results

### Scale Reliability

Scale reliability for the five subscales of MFQ20 and MFSS is calculated using Cronbach’s alpha (Table 1). Internal consistency of the subscales for MFQ20 produces low Cronbach’s alpha coefficients: .47 (Care), .41 (Fairness), .51 (Loyalty), .53 (Authority), and .50 (Sanctity). Alphas for each foundation in MFSS are .71 (Care), .63 (Fairness), .59 (Loyalty), .70 (Authority), and .53 (Sanctity). Thus, five subscales for MFQ20 and four subscales for MFSS do not surpass what is generally considered an acceptable Cronbach’s alpha cutoff (a Cronbach’s alpha > .70 is a widely used rule of thumb in social studies (Nunnally & Bernstein, 1994)).

**Table 1:** Cronbach’s alpha coefficients for the five subscales of MFQ-20 and MFSS.


	MFQ-20 CRONBACH’S α (4 ITEMS/FOUNDATION)	MFSS CRONBACH’S α (4 ITEMS/FOUNDATION)

Care/Harm	.47	.71

Fairness/reciprocity	.41	.63

Loyalty/betrayal	.51	.59

Authority/respect	.53	.70

Sanctity	.50	.53

FULL SCALE (20 items)	.70	.86


### Confirmatory Factor Analysis

For both MFQ20 and MFSS, several theoretical factor structures are tested. Inspired by Graham, Haidt, and Nosek (2009) and Graham *et al*. (2011), in the first step, first-order models are compared. It is hypothesized that a five correlated factors model would provide a better overall model fit than a single morality factor model, a two-factor model representing Individualizing and Binding foundations, or a three-factor model corresponding to Shweder *et al*.’s (1997) account of three ethics of Autonomy (Care/Fairness), Community (Loyalty/Authority) and Divinity (Sanctity). Table 2 presents the results of the Confirmatory Factor Analysis among the Moral Foundations models. For the MFQ20, the hypothesized five-factor model fails to converge due to highly correlated factors of Fairness and Purity. The three-factor model provides moderately acceptable model fit indices for the MFQ20: WLSMVχ^2^ = 2348.28, *df* = 167; *p* < .001; CFI/TLI = .720/680; RMSEA = .081; WRMR = 2.98. This model is retained for further measurement invariance testing in MFQ20, despite poor CFI/TLI values. For the MFSS, the overall best model is the five-factor model with the following fit indices WLSMVχ^2^ = 1126.86, *df* = 160; *p* <.001; CFI/TLI = .940/.930; RMSEA = 0.06; WRMR = 1.58. This model is retained for further measurement invariance testing in MFSS. In sum, CFAs do not provide support for the proposed five-factor model in MFQ20. A three foundations model in the MFQ20 is retained, despite poor fit indices (CFIs below .90). In contrast, model fit indices for the taboo trade-off items of MFSS are acceptable in the five-factor model.

**Table 2:** Results of Confirmatory Factor Analysis for the relationships among Moral Foundations models.


MODEL	*WLSMVχ* ^2^	*df*	*CFI/TLI*	RMSEA	WRMR

*MFQ20*					

**A.** One-factor model	3907.90***	170	.520/.470	.105	3.84

**B.** Two-factors model	2887.28***	169	.650/.610	.090	3.32

**C.** Three-factors model	2348.28***	167	.720/.680	.081	2.98

**D.** Five-factors model	*The model fails to converge*				

*MFSS*					

**A.** One-factor model	1838.63***	170	.900/.890	.070	2.09

**B.** Two-factors model	1426.37***	169	.930/.920	.060	1.83

**C.** Three-factors model	1346.65***	167	.930/.920	.060	1.76

**D.** Five-factors model	1126.86***	160	.940/.930	.060	1.58


*Note*: Structural equation modeling was used for the analyses. Weighted Least Squares Means and Variances (WLSMV) was used for model estimation. CFI = comparative fit index; TLI = Tucker Lewis Index; RMSEA = root-mean-square error of approximation; WRMR = weighted mean-square residual standardized. *** *p* <.001. In each *Model A*, respectively, the 20 items of MFQ, and MFSS are loaded into one factor. In each *Model B*, Care, and Fairness items are loaded onto one factor, Loyalty, Authority, and Sanctity items are loaded onto a second factor. In each *Model C*, Care and Fairness items are loaded onto one factor, Loyalty and Authority factors are loaded onto a second factor, and Sanctity items are loaded onto a third factor. In each *Model D*, Care items are loaded onto one factor, Fairness items are loaded onto a second factor, Loyalty items are loaded onto a third factor, Authority items are loaded onto a fourth factor, and Sanctity items are loaded onto a fifth factor.

### Correlations among and between MFQ20 and MFSS

**Figure 2:**
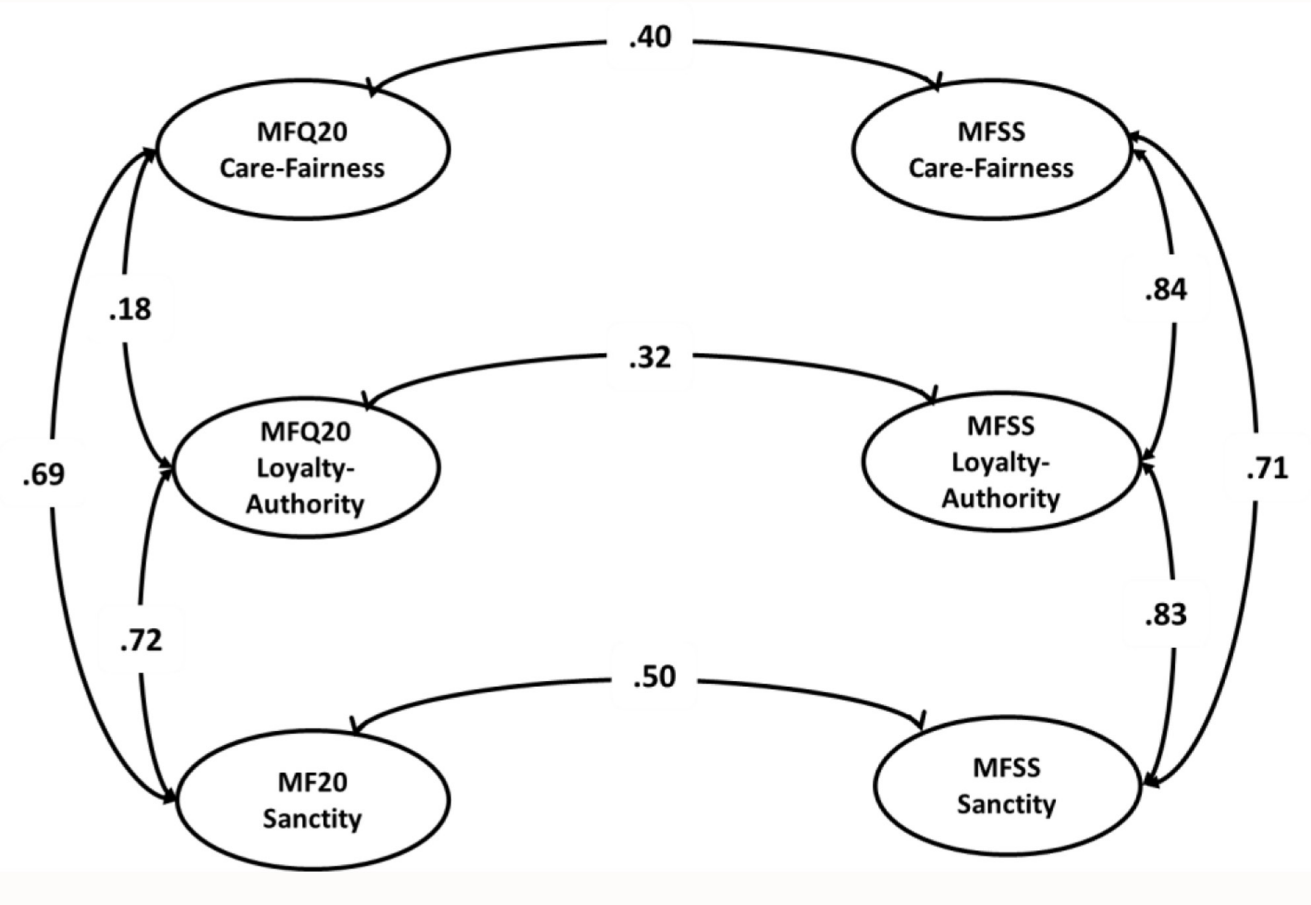
Pearson correlations among and between MFQ20 and MFSS three-factor model. *Note*: Figure 2 represents Pearson correlations between and among MFQ20 and MFSS’ three-factor model Correlations among MFQ20 subscales are represented on the left, and correlations among MFSS subscales are on the right. For the sake of clarity and parsimony, correlations between MFQ20 and MFSS subscales are represented for their counterparts only. Correlations between non-equivalent subscales of both questionnaires are found in Table 3. CFA-model fit indices: WLSMVχ^2^ = 4365.65, *df* = 725; χ^2^/*df* = 6.02; RMSEA = 0.05; WRMR = 2.28; CFI/TLI = 0.86/0.85.

Correlation coefficients are estimated within a CFA framework. Because the five-factor model does not converge in the MFQ20, the correlations of the three-factor models (Care/Fairness, Loyalty/Authority, and Sanctity) in both surveys are estimated. Latent modeling is chosen because latent analysis takes measurement error into account, which is not the case in a manifest model. Correction for measurement error leads to a disattenuation of the relationships at the latent level (Geiser, 2013). Standardized correlations among the subscales of each questionnaire are visualized in Figure 2. In the MFQ20, the correlation between Care/Fairness and Loyalty/Authority is moderate (*r* = .18), and correlations between Loyalty/Authority and Sanctity and Care/Fairness and Sanctity are high (resp. *r* = .72 and *r* =.69). In MFSS, all correlations between the subscales are high, ranging from *r* = .71 (correlation between Care/Fairness and Sanctity) to *r* = .84 (correlation between Care/Fairness and Loyalty/Authority). Subscales Care/Fairness, Loyalty/Authority, and Sanctity are moderately correlated with their MFSS counterparts (Figure 2) and all other MFSS subscales (Table 3), except for a nonsignificant correlation between subscales Care/Fairness and MFQ20 Loyalty/Authority.

**Table 3:** Standardized correlations between MFQ20 and MFSS subscales.


MFQ-20 SUBSCALES THREE-FACTOR MODEL	MFSS SUBSCALES THREE-FACTOR MODEL		

	F1 CARE FAIRNESS	F2 LOYALTY AUTHORITY	F3 SANCTITY

**F1 = CARE FAIRNESS**	.40***	.21***	.26***

**F2 = LOYALTY AUTHORITY**	Ns	.32***	.31***

**F3 = SANCTITY**	.23***	.34***	.50***


*** *p* < .001; ns = not significant.

Both MFQ20 and MFSS questionnaires were designed to measure the same moral foundations. On the one hand, some degree of correspondence may be expected between the corresponding subscales. Indeed, correlation coefficients are moderately high with the highest correlation between the MFQ20 Sanctity subscale and its counterpart in MFSS (*r* = .50). On the other hand, a very strict match between both surveys’ subscales is not expected because MFQ20 and MFSS are dissimilar in that the former contains two response formats, whereas the latter measures indirectly how much participants value each of the five moral foundations by asking how much money it would take for someone to commit actions that violate each of these foundations. The idea behind the MFSS scale is to see whether answers on the sacredness scale reveal the *same general pattern* as answers on the MFQ. Thus, a certain degree of convergence between the questionnaires’ corresponding subscales is expected but not a very strict match because endorsement of each of the five moral foundations is measured differently.

### Testing measurement invariance across gender

Measurement invariance (MI) is the statistical property of a measurement that indicates that the same underlying factor (unobserved variable) is being measured across groups (or across time). MI is evidenced when the relationships between indicator variables (manifest variables) and the underlying factor are the same across groups. When MI is evidenced, group comparisons are meaningful. The same factor is measured across groups, and group differences reflect true group differences in the variables of interest. When MI is not evidenced, analyses of the corresponding measures do not produce meaningful results. Findings of differences between groups cannot be unambiguously interpreted because observed group differences cannot be assumed to be accurate (Byrne, 2003; Horn & McArdle, 1992; Meredith, 1993). MI testing involves a sequence of four hierarchical, increasingly restrictive models (1) configural invariance, (2) weak MI, (3) strong MI, and (4) strict MI (Meredith, 1993). In this section, we present the results of the MI tests to evidence if the same *three-factor model* for MFQ20 and the same *five-factor model* for MFSS, holds across males and females.

#### MFQ20

Firstly, we test whether the three-factor baseline model provides a good fit for both males and females separately. Model fit results (not shown here) are poor for both females (*N* = 1062) (WLSMVχ^2^ = 1443.66, *df* = 167; CFI/TLI = .700/.660; RMSEA = .09; WRMR = 2.34) and males (*N* = 435) (WLSMVχ^2^ = 649.52, *df* = 167; CFI/TLI = .730/.690; RMSEA = .08; WRMR = 1.60). Identical baseline models are retained, for males and females, with the same three-factor structure and with the same pattern of fixed and free factor loadings. Model fit is not improved based on the modification indices. Once a baseline model is determined for both groups, they are integrated into a multi-group CFA model – a configural model – that is implemented simultaneously across both groups (Byrne, 2012; Horn & McArdle, 1992; Vandenberg & Lance, 2000). Secondly, a multi-group CFA is used to see whether the factor structure holds equal across the two groups. Table 4 presents the results of the MI tests for the MFQ20 three-factor model. In the configural model, the same number of factors and the same pattern of fixed and free factor loadings are specified in each of the groups. WLSMV estimation of the configural model for gender yields the following goodness-of-fit statistics: WLSMVχ^2^ = 2051.68; *df* = 334; *p* < .001; RMSEA = .083; 90%CI = [.079, .086]; WRMR = 2.84; CFI/TLI = .702/.661. Although the fit statistics of the configural or pattern invariance model are only moderately acceptable (poor CFI/TLI, fair fit RMSEA), the fit of this configural model provides the baseline value against which the first comparison of models is made. No modification indices are allowed to achieve a better model fit. The second model represents the weak measurement invariance model; defined as the invariance of factor loadings across groups. If factor loadings are invariant across groups, then measures across groups are considered to be on the same scale (Wang & Wang, 2020). The DIFFTEST is utilized for difference testing between the restricted model (in which factor loadings are set equal across groups) and the unrestricted configural model (in which there are no constraints). Goodness-of-fit statistics related to the factor-loading invariant model (weak invariance model) are WLSMVχ^2^ = 1965.68; *df* = 334; *p* < .001; RMSEA = .078; 90%CI = [.075, .082]; WRMR = 2.91; CFI/TLI = .720/.696. ΔCFI = –.002; DIFFtest: ΔWLSMVχ^2^ = 54.16, with 17 degrees of freedom and a probability of less than .001 (*p* < .001). With a significant *p*-value, the null hypothesis of factor loading invariance must be rejected, meaning that factor loadings are not significantly different between females and males samples. This implies that the instrument under study is potentially problematic because it may measure different factors in different populations. As a result, no further invariance testing is necessary. When full measurement invariance is not achieved, some researchers suggest that partial measurement invariance may be tested and comparisons of groups on latent variables can be conducted if partial measurement invariance holds (Byrne, Shavelson, & Muthén, 1989). One could identify and delete the problematic items, and then re-test the weak invariance hypothesis using a subset of the original items. However, both approaches are debatable (Wang & Wang, 2020).

**Table 4:** Results of measurement invariance of the three-factor model: MFQ20 items (N_females = 1062; N_males = 435).


MODEL	WLSMVχ^2^			RMSEA			WRMR	CFI/TLI	ΔCFI	DIFFtest Δχ^2^ (Δ*df*)
	
	VALUE	*df*	*p*	VALUE	95%CI	*p*				

Configural model	2051.68	334	<.001	.083	[.079, .086]	.000	2.84	.702/.661		

Weak invariance	1965.53	351	<.001	.078	[.075, .082]	.000	2.91	.720/.696	.02	54.16 (17) *p* < .001


#### Taboo trade-off items of MFSS

In line with the procedure described above, we establish a baseline model by testing whether the five-factor model in MFSS provides a good fit for both males and females separately. Model fit results (not shown here) are good for both females (*N* = 1062) (WLSMVχ^2^ = 594.28, *df* = 160; CFI/TLI = .960/.950; RMSEA = .05; WRMR = 1.20) and males (*N* = 435) ((WLSMVχ^2^ = 441.36, *df* = 160; CFI/TLI = .930/.910; RMSEA = .06; WRMR = 1.06). The same five-factor model is supported in both groups. Next, a configural model is estimated simultaneously with the two groups. Table 5 presents the results of the MI tests. WLSMV estimation of the configural model for gender yields the following goodness-of-fit statistics: WLSMVχ^2^ = 1018.20; *df* = 320; *p* < .001; RMSEA = .054; 90%CI = [.050, .058]; WRMR = 1.60; and CFI/TLI = .946/.936. Fit statistics of the configural model are acceptable. Next, the weak invariance hypothesis is tested. Goodness-of-fit statistics related to the factor-loading invariant model are WLSMVχ^2^ = 909.48; *df* = 335; *p* < .001; RMSEA = .048; 90%CI = [.044, .052]; WRMR = 1.66; CFI/TLI = .956/.950. Comparison of the relative fit of the constrained model (weak invariance model) with the relative fit of the configural model yields the following results: DIFFtest: ΔWLSMVχ^2^ = 22.55, with 15 degrees of freedom and a probability of *p* =.094. A non-significant *p*-value indicates that the weak invariance hypothesis is retained. Next, strong invariance is tested by imposing equality constraints on thresholds. Goodness-of-fit statistics related to the strong invariant model are WLSMVχ^2^ = 1244.73; *df* = 446; *p* < .001; RMSEA = .049; 90%CI = [.046, .052]; WRMR = 2.01; CFI/TLI = .940/.950. Comparison of the relative fit of the constrained model (strong invariance model) with the relative fit of the weak invariance model yields the following results: DIFFtest: ΔWLSMVχ^2^ = 379.08, with 111 degrees of freedom and a probability of less than .001 (*p* < .001). A significant *p*-value indicates that the strong invariance model cannot be retained, meaning that its correspondence to the data is worse than that of the weak invariance model. Further invariance testing is not necessary.

**Table 5:** Results of measurement invariance of the five-factor model: MFSS (N_females = 1060; N_males = 436).


MODEL	WLSMVχ^2^			RMSEA			WRMR	CFI/TLI	ΔCFI	DIFFtestΔχ^2^ (Δ*df*)
	
	VALUE	*df*	*p*	VALUE	95% CI	*p*				

Configural model	1018.20	320	<.001	.054	[.050, .058]	.038	1.60	.946/.936	–.01	

Weak invariance	909.48	335	<.001	.048	[.044, .052]	.821	1.66	.956/.950	.02	22.55 (15) *p* < .094

Strong invariance	1244.73	446	<.001	.049	[.046, .052]	.703	2.01	.940/.950		379.08 (111) *p* < .001


In sum, we tested measurement invariance across gender of the three-factor model in MFQ20 and of the five-factor model in MFSS. It is found that males and females show the same factor pattern in both questionnaires, despite poor fit indices for MFQ20. Weak invariance hypothesis is retained for MFSS but not for MFQ20. This means that the three-factor structure of the MFQ20 is not invariant over the two groups at the level of factor loadings. However, retention of the weak invariance model of MFSS supports the claim that the five-factor structure of the MFSS is manifested in the same way in each group. Implications are that variances and covariances at the latent level can be formally compared across groups (Kline, 2016).

## Discussion

Moral Foundations Theory (Haidt & Joseph, 2004) has drawn a lot of attention from researchers across the world. To test MFT’s multi-foundational propositions, two quantitative self-report instruments are developed: the Moral Foundations Questionnaire (MFQ) and the Moral Foundations Sacredness Scale (MFSS). The present study set out to test the generalizability of MFT’s hypothesized five foundations structure in the two quantitative questionnaires (a 20-item short version of the original MFQ (MFQ20) and the original 20-item MFSS) using the *Dutch translations* in a student sample (*N* = 1496).

Our first goal was a comparison of several first-order measurement models. We hypothesized to find the same five-factor structure as posited by MFT. Results show that the five-factor model holds in the MFSS but is problematic in the MFQ20. The results suggest that five foundations in the latter questionnaire are too many for the data: we found very high correlations between the Sanctity and Fairness foundations, several cross-loadings (one item loading on different factors), and correlated error structures (due to items that may be interpreted differently across gender) (Lubke & Dolan, 2003). These findings may be due to the fact that the abridged version of MFQ (20 items) is used. Possibly the results might have been different with more items. However, when developing the MFQ, Graham *et al*. (2011) quantified how the quality of the scale would decline if shortened. Their initial analyses revealed that the optimal two-item combinations were nearly as good as the three-item combination. Although it was suggested that the latter is preferable for their broader conceptual coverage. Indeed, using the longer version of MFQ (30 items), a set of studies found support for the five-factor model over alternative models (as was the case of the original study (Graham *et al.*, 2011)), although the model fit of the five-factor model was not optimal (see also Nilsson & Erlandsson, 2015; Yilmaz *et al.*, 2016). Other studies, on the other hand, failed to identify the proposed five-factor structure (Akhtar, Francis, Village *et al.*, 2023; Iurino & Saucier, 2020). We’ll come back to this issue later. In the present study, a three foundations model, representing the “big three of morality” (Shweder *et al.*, 1997) was retained in MFQ20 for further measurement invariance testing, despite low fit criteria (low CFI/TLI). The five foundations model was retained in MFSS.

Our second goal was a comparison of correlations among and between MFQ20 and MFSS subscales within a CFA framework. As expected, a certain degree of convergence between equivalent subscales is found. However, we did not find a very strict match because both surveys focus on different aspects. Of particular interest are participants’ answers on both surveys and the extent to which the same general patterns hold across different ways of measuring moral values (YourMorals.org). Correlations among MFSS subscales are all high. These high correlations may be due to the provocative nature of the items. Participants may experience more resistance when confronted to perform taboo actions for some amount of money (see also Yalçındağ *et al.*, 2019).

Finally, our third goal was to test the measurement invariance (MI) of the two moral foundations questionnaires within a CFA framework across gender. The analyses of data from males and females were performed based on the five-factor model of MFSS and on the three-factor model of MFQ20 The weak invariance hypothesis was retained for the MFSS but not for the MFQ20. The former suggests that the factor loadings of the items are equivalent across gender and that the latent constructs measured by MFSS have the same meaning to males and females. Weak (or factorial) invariance implies that the regression slopes are equivalent across the groups. Attaining weak invariance suggests that group comparisons of factor variances and covariances are defensible, however, the comparison of group means is not justified at this level (Byrne, 2003; Meredith & Teresi, 2006; Vandenberg & Lance, 2000). Failing to evidence MI for the MFQ20 suggests that the latent constructs, as measured by MFQ20, are not equivalent across males and females meaning that they have a different structure or meaning to males and females. Thus, the constructs cannot be meaningfully compared across the two groups, which is problematic. For instance, if Fairness items would load strongly on the Fairness foundation in one group, but weakly in another group because of different interpretations of the items, then the Fairness factor would have different meanings to males and females (the factor would not be equivalent or invariant) and between-groups comparison of mean scores would not be valid. Consequently, ensuring equivalence of the meaning of a latent construct between groups is a prerequisite to making valid comparisons of subgroup means (Putnick & Bornstein, 2016).

To summarize, (1) CFA based on data collected with the original 20-item MFSS yields an acceptable model fit for the proposed five-factor structure whereas the five-factor model fails to converge unproblematically based on analyses of the 20-item short version of the original MFQ; (2) correlations between MFSS subscales are very high; and (3) weak MI is evidenced in MFSS but not in MFQ20.

In the remainder of this section, we further elaborate on the evidence, presented here and previously, for the validity of the quantitative measures of Moral Foundations Theory. Indeed, measurement issues have previously been the subject of critiques on MFT’s quantitative self-report questionnaires. In what follows, we particularly focus on MFQ because this is the most widely used measure. Theoretical and methodological critiques of MFT have called into question the psychometric properties of the MFQ questionnaire. The critiques can be categorized under three headings (1) internal consistency of each moral foundation cluster is below the conventional thresholds; (2) the validity of the posited five-factor model provides a poor fit to the data, and (3) measurement non-invariance across groups preclude meaningful comparisons across populations because patterns of responding differ from one population to another.

*Firstly, internal consistency*. Across the literature on MFT and MFQ in particular, there have been concerns about overall low values of the subscales’ reliability (indicated by Cronbach’s alpha). In the present study, Cronbach’s alpha for both questionnaires’ subscales are poor: for MFQ20 (all alpha’s less than .53) but slightly better for MFSS (lowest .53, highest .71). Poor Cronbach’s alphas have been reported in other studies (e.g. Davis, Rice, Van Tongeren *et al.*, 2016; Harper & Hogue, 2019). In a systematic examination of 530 empirical studies using MFQ, Tamul *et al*. (Tamul, Elson, Ivory, Hotter, Lanier *et al.*, 2020) analyzed the reported reliabilities. For 61% (*n* = 210) of the samples, Cronbach’s alpha was reported as a measure of reliability. Results suggested that the average Cronbach’s alpha scores for four of the five foundations were below .70. The MFQ20 short version tended to produce even lower values than the MFQ30 long version. Weighting the scores by sample size did not change the average alpha; the value of alpha either remained the same or slightly increased. Graham *et al*., (2009,2011) report reliability performances (indicated by Cronbach’s alpha) of the MFQ30 subscales between .39 and .70, also below what is generally considered as an acceptable threshold (Nunnally & Bernstein, 1994). From the point of view of the authors of MFQ, internal consistency was argued as important but so was comprehensive coverage of the theoretical constructs (Graham *et al.*, 2011). In scale development, instead of maximizing alpha, the authors of MFQ aimed to achieve a trade-off between sufficient internal consistency and maximal item heterogeneity to increase confidence that the foundation is fully represented (p. 370). However, Tamul *et al*. (2020) have argued that Cronbach’s alpha has been the subject of criticism as an inappropriate measure for scale reliability and encourage MFT researchers to include alternative indicators of the psychometric properties of the MFQ subscales such as McDonald’s omega.

*Secondly, the five-factor structure*. When using MFT’s default five-factor model in CFA, low to moderately acceptable levels of fit indices are found in different studies (CFI < .90, RMSEA > .08) (e.g. Davis, Rice, Van Tongeren, Hook, DeBlaere *et al.*, 2016; Iurino & Saucier, 2020; Nilsson & Erlandsson, 2015), calling the utility of the five-factor structure of the MFQ in question. For instance, Iurino and Saucier (2020) based their analyses on the 20-item short version of MFQ and found no evidence for the proposed five-factor model across diverse populations. Possible explanations for this finding were suggested, (1) the use of less motivated and smaller samples compared to the samples used in the original study of Graham *et al*. (2011), (2) different administration modes of the questionnaires (fixed item order); and (3) use of the short-form MFQ with only two indicators per factor which might have been too few for the suggested five foundations. The authors suggest examining other factor structures that would be more robust across cultures, such as a model where each foundation maps onto a distinct emotion or one where each foundation maps onto a specific function. Recently, attempts are made to remap the moral foundations’ structure into a better-fitting model of the MFQ. For example, Harper and Rhodes (2021) re-examined and revised the initial five-factor structure of the MFQ30 (and the extended six-factor structure combining the initial MFQ with Liberty items (see Iyer *et al.*, 2012). Using different analytical techniques (EFA, CFA, and network analysis), the authors present a theory-driven three-factor model, labeled ‘Traditionalism’ – ‘Compassion’ – and ‘Liberty’ that demonstrates a better fit to the data compared to the initial five-factor structure (including superior internal consistency coefficients) However, Harper and Rhodes’ revised MFQ should be viewed as exploratory in nature and should be further confirmed cross-culturally (exclusively British samples were used). Zakharin and Bates (2021) constructed a seven-foundation model in which the two individualizing foundations (harm/care and fairness/reciprocity foundations) are preserved and in which two new foundations are added to the original three binding foundations (clan loyalty, country loyalty, hierarchy, sanctity, and purity). This seven-foundation model has been tested and validated in data collected from the UK, US, Australia, and China, providing a good fit to the data in all four samples. However, Nilsson (2023) points out that, compared to the original moral foundations model, this new multi-foundational model is not theory-driven but rather data-driven. In contrast, alternative theory-based measures to MFQ are proposed by Curry, Chesters, and Van Lissa (2019) (Morality-as-Cooperation questionnaire based on MFQ) and by Leitgöb, Eifler, and Weymeirsch (2020) (ALLMOR, an instrument to capture general moral concepts based on MFQ). These fine-grained alternative questionnaires to conceptualize the moral domain require further validation studies.

*Thirdly, measurement invariance*. As already mentioned above, the present study was unable to find support for measurement equivalence of the five-factor model in MFQ20 across gender, ruling out meaningful comparisons between males and females. Results of measurement invariance tests in other studies are mixed. In a wide cross-national, cross-cultural sample (27 countries) Iurino and Saucier (2020) were unable to evidence MI of the short version MFQ20. Overall, the results indicated that the five-factor structure resulted in nonpositive-definite matrices in most countries, due to highly correlated factors, and too many factors for the data. In contrast, Davies, Sibley, and Liu (2014) were able to establish weak invariance across gender in a large New Zealand sample utilizing the MFQ30 (long version). Similarly, Davis *et al.*, (2016) tested MI of the MFQ in US Black and White samples and were able to evidence weak (or metric) invariance. In addition, Atari, Lai, and Dehghani (2020) provided a large-scale examination of moral foundations, as measured by MFQ30 (long version), and provided comprehensive evidence that sex differences in the pattern of moral judgments can be meaningfully compared across cultures. Also, recently, Nilsson (2023) found acceptable measurement invariance of the original MFQ across sex, based on the five-factor model (among other more complex models). Furthermore, Andersen, Zuber, and Hill (2015) also used the MFQ30 in a sample of business students but found that the overall five-factor model did not hold across males and females. However, partial metric invariance was sought by systematically eliminating some of the factor loading constraints across both groups. Partial measurement invariance is described as an intermediate state of invariance by Byrne, Shavelson, and Muthén (1989), although there are no clear guidelines for determining the degree of partial invariance that would be acceptable for concluding that indicators measure approximately the same things over groups (Kline, 2016). Meuleman and Billiet (2012) argue that meaningful comparisons can be made if equivalence holds for at least two items per construct. In the present study, a *standard CFA factor structure* is assessed in which each indicator is specified to load on only one factor and not on another factor (no cross-loading items allowed), and in which measurement error is not correlated with other measurement errors. No post hoc modifications were allowed to improve model fit. It is not uncommon to find that the fit of a proposed model is poor. However, allowing modifications to the proposed model to find an acceptable fit to the data, based on modification indices, should be done only when the modifications are theory-driven (MacCallum, 1995). In addition, post hoc modifications to improve model fit may capitalize on chance variations in the sample and any such modifications should be viewed as explorative until cross-validated on other samples (Jackson, Gillaspy & Purc-Stephenson, 2009). Wang and Wang (2020) argue that cross-loading items are an undesirable feature of a measurement instrument because they lead to a complex factor structure that is difficult to cross-validate (p. 42). Strategies to deal with deviations from measurement invariance are suggested by Van De Schoot et al. (2015). Because we aimed to cross-validate the Dutch translation of MFQ20 (and MFSS) we devoted our attention solely to detecting the presence/absence of invariance of the best-fitting CFA model. According to Meuleman and Billiet (2012), a lack of measurement equivalence should not lead to precluding substantive analysis. One can still perform cross-cultural, cross-national analyses and look for broad patterns of similarities or divergences in relations between countries, even when only configural invariance is found.

Notwithstanding, in response to the theoretical critiques of MFT and the psychometric findings of the MFQ in diverse samples, the authors of the original MFQ developed the Moral Foundations Questionnaire-2 (MFQ-2) (Atari, Haidt, Graham *et al.*, 2022). MFQ-2 is theoretically refined and psychometrically improved: the four original foundations of Care, Loyalty, Authority, and Purity are retained while the Fairness foundation is replaced by two distinct foundations of Equality and Proportionality (an argument already elaborated on by Haidt in his book *The righteous mind: Why good people are divided by politics and religion* (2013)). So far, this new MFQ-2 is validated by the authors across three studies using data from 25 populations (including Belgium) and presented as *… a psychometrically superior and truly cross-cultural and cross-linguistic instrument…* (p. 61). In short, MFQ-2 is presented as a more accurate instrument for mapping and investigating the network of moral foundations cross-culturally.

## Conclusion

The Moral Foundations Questionnaire and the Moral Foundations Sacredness Scale are quantitative self-report measures Both scales have been used in a wide variety of empirical studies and across cultures to assess the utility and accuracy of MFT’s multi-foundational propositions. Examination and cross-validation of the measures are fundamental to the precision and validity of *“estimates underlying inferences on the development of and differences in moral morality”* (Tamul *et al.*, 2020: 2). Thus, it is critical that the questionnaires have robust psychometric properties. Overall, the present study shows that the MFSS scale performs better than the MFQ20 in terms of scale reliability, fit indices, and measurement invariance testing. However, given that the Moral Foundations Sacredness Scale has been used to a much lesser extent and, as a result, received much less methodological scrutiny compared to MFQ, more methodological inquiries on MFSS are welcomed. As for the MFQ20, it is safe to conclude that its use should be discouraged altogether, because, as previously also argued by proponents of MFT, it is hard to get a good and reliable measure with just four items for each foundation (MoralFoundations.org). Instead, the authors of the Moral Foundations Theory (Atari, Haidt, Graham, *et al.*, 2022) recently developed a revised version of MFQ (MFQ-2) as a more accurate instrument for the conceptualization of morality. The extent to which this revised measurement instrument responds to the many (theoretical and methodological) criticisms and possesses adequate psychometric properties will have to be empirically tested in further research. Researchers in Dutch-speaking populations are strongly encouraged to take up the challenge.

## Additional File

The additional file for this article can be found as follows:

10.5334/pb.1188.s1 Appendices. Appendix 1–7.

## Footnotes

Henrich, Heine and Norenzayan (2010) proposed that the majority of research samples used in mainstream behavioral studies are characterized by participants from Western, Educated, Industrialized, Democratic, and Rich (WEIRD) cultures. However, these WEIRD people represent only a small minority in the world, rendering them particularly unusual compared to other populations.
