# Supplementary material for: Moral Foundations Questionnaire and Moral Foundations Sacredness Scale: Assessing the Factorial Structure of the Dutch Translations
Source: Psychol Belg. 2023 Jul 24;63(1):92–104. doi: 10.5334/pb.1188 (PMC10376904; doi:10.5334/pb.1188)
Supplement: Appendices. — Appendix 1–7. [file pb-63-1-1188-s1.pdf]

APPENDIX 1

Table 1

Descriptives

	HUMAN COOPERATION-survey (2019)	Frequency	Valid Percent
GENDER			
	Female	1061	70.9%
	Male	435	29.1%
AGE	18y or younger	577	38.6%
	19-20y	458	30.6%
	21y or older	461	30.8%
FACULTY	Faculty of Arts and Philosophy	156	10.4%
	Faculty of Law and Criminology	674	45.1%
	Faculty of Sciences	55	3.7%
	Faculty of Medicine and Health Sciences	89	6.0%
	Faculty of Engineering and Architecture	58	3.9%
	Faculty of Economics and Business Administration	162	10.8%
	Faculty of Veterinary Medicine	62	4.1%
	Faculty of Psychology and Educational Sciences	111	7.4%
	Faculty of Bioscience Engineering	35	2.3%
	Faculty of Pharmaceutical Sciences	7	0.5%
	Faculty of Political and Social Sciences	85	5.7%

APPENDIX 2

MORAL FOUNDATIONS QUESTIONNAIRE: 20-Items Short Version

Part 1. Moral relevance items (responded to using the following response options: not at all relevant, not very relevant, slightly relevant, somewhat relevant, extremely relevant)

MATH – Whether or not someone was good at math (this item is not scored; it is included both to force people to use the bottom end of the scale and to catch and cut participants who respond with 3 or above)

When you decide whether something is right or wrong, to what extent are the following considerations relevant to your thinking? Please rate each statement using this scale:

Harm/care

EMOTIONALITY - Whether or not someone suffered emotionally

WEAK - Whether or not someone cared for someone weak or vulnerable

Fairness/reciprocity

TREATED - Whether or not some people were treated differently than others

UNFAIRLY - Whether or not someone acted unfairly

Loyalty/loyalty

LOVECOUNTRY - Whether or not someone's action showed love for his or her country

BETRAY - Whether or not someone did something to betray his or her group

Authority/respect

RESPECT - Whether or not someone showed a lack of respect for authority

TRADITIONS - Whether or not someone conformed to the traditions of society

Sanctity/sanctity

DECENCY - Whether or not someone violated standards of Sanctity and decency

DISGUSTING - Whether or not someone did something disgusting

Part 2. Moral relevance items (responded to using the following response options: strongly disagree, moderately disagree, slightly disagree, slightly agree, moderately agree, strongly agree)

GOOD – it is better to do good than to do bad (this item is not scored, it is included to force use of top of the scale, and to catch and cut people who respond with first 3 response options)

Please read the following sentences and indicate your agreement or disagreement

COMPASSION – Compassion for those who are suffering is the most crucial virtue

ANIMAL – One of the worst things a person could do is hurt a defenseless animal

Fairness/reciprocity

FAIRLY – When the government makes laws, the number one principle should be ensuring that everyone is treated fairly

JUSTICE – Justice is the most important requirement for a society

Loyalty/loyalty

HISTORY – I am proud of my country's history

FAMILY – People should be loyal to their family members, even when they have done something wrong

Authority/respect

KIDRESPECT – Respect for authority is something all children need to learn

SEXROLES – Men and women each have different roles to play in society

Sanctity/sanctity

HARMLESSDG – People should not do things that are disgusting, even if no one is harmed

UNNATURAL – I would call some acts wrong on the grounds that they are unnatural

APPENDIX 3

<u>MORAL FOUNDATIONS SACREDNESS SCALE: 20-Items Version</u>

Created by Jesse Graham and Jonathan Haidt

March 5, 2010

Instructions: Try to imagine *actually doing* the following things, and indicate how much money someone would have to pay you, (anonymously and secretly) to be willing to do each thing. For each action, assume that nothing bad would happen to you afterwards. Also assume that you cannot use the money to make up for your action.

Scale: 1) $0 (I'd do it for free) - 2) $10 - 3) $100 - 4) $1000 - 5) $10,000 6) $100,000 - 7) NEVER for any amount of money

Harm

DOGKICK: Kick a dog in the head, hard

ENDANGERED: Shoot and kill an animal that is a member of an endangered species

OVERWEIGHT: Make cruel remarks to an overweight person about his or her appearance

PALM: Stick a pin into the palm of a child you don't know

Fairness

CARDS: Cheat in a game of cards played for money with some people you don't know well

APARTMENT: Say no to a friend's request to help him move into a new apartment, after he helped you move the month before

BALLOTS: Throw out a box of ballots, during an election, to help your favored candidate win

RACEPLEDGE: Sign a secret-but-binding pledge to only hire people of your race in your company

Loyalty

TALKRADIO: Say something bad about your nation (which you don't believe to be true) while calling in, anonymously, to a talk-radio show in a foreign nation

FAMILYSHUN: Break off all communications with your immediate and extended family for 1 year

CITIZENRENOUNCE: Renounce your citizenship and become a citizen of another country

LEAVECLUB: Leave the social group, club, or team that you most value

Authority

PARENTCURSE: Curse your parents, to their face. (You can apologize and explain one year later)

HANDGESTURE: Make a disrespectful hand gesture to your boss, teacher, or professor

ROTTENTOMATO: Throw a rotten tomato at a political leader you dislike. (remember, you will not get caught)

FATHERSLAP: Slap your father in the face (with his permission) as part of a comedy skit

Sanctity

SOULSELL: Sign a piece of paper that says “I hereby sell my soul, after my death, to whoever has thispiece of paper”

TAIL: Get plastic surgery that adds a 2 inch tail on to the end of your spine (you can remove it in three years)

MOLESTERBLOOD: Get a blood transfusion of 1 pint of disease-free, compatible blood from a convicted child molester

STAGEANIMAL: Attend a performance art piece in which all participants (including you) have to act like animals for 30 minutes, including crawling around naked and urinating on stage

Nonmoral (optional)

ICEBATH: Sit in a bathtub full of ice water for 10 minutes

IDIOTSIGN: Wear a sign on your back for one month that says, in large letters, “I am an idiot.”

HEADACHE: Experience a severe headache for two weeks

LOSEHEARING: Lose your sense of hearing for one year

APPENDIX 4

Figure 1

Competing Model 1: Single factor model. Model is shown for the MFQ-20 and 20-item MFSS

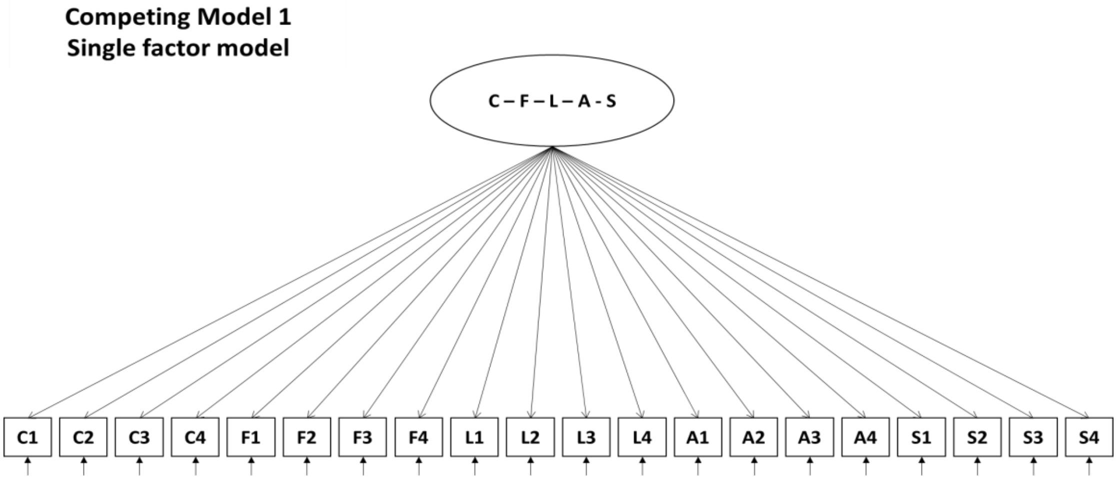

Note: Model 1 represents a single factor structure in which H1 till P4 are 20 observed indicators that load on a single factor (C – F – L – A – S; where C = Care – F = Fairness – L = Loyalty – A = Authority and S = Sanctity).

APPENDIX 5

Figure 2

Competing Model 2: Two correlated factors model. Model is shown for the MFQ-20 and 20-item MFSS

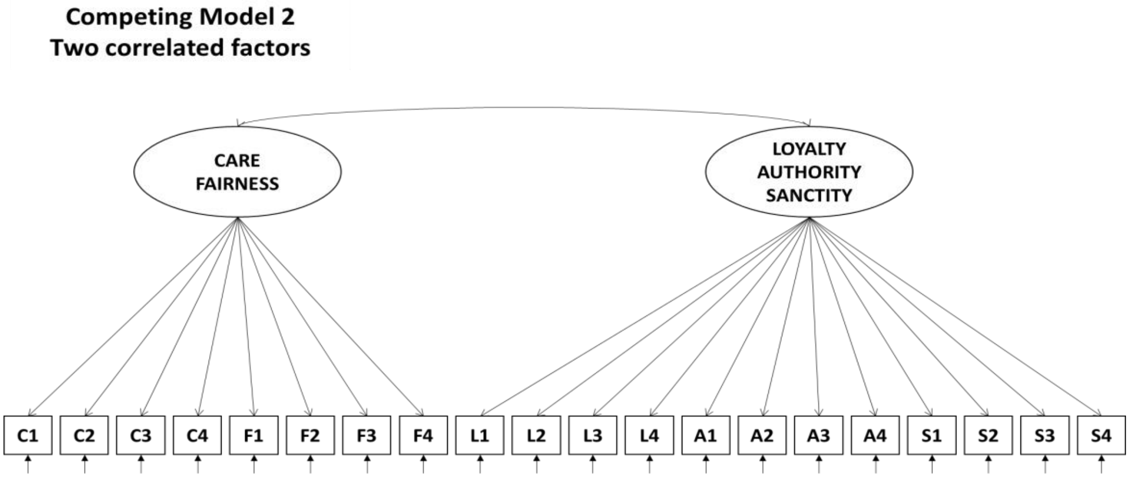

Note: Model 2 represents a two-correlated factor structure in which the two factors are jointly modeled and the relationship between the factors is estimated. Factor Care-Fairness has 8 indicators (C1 till F4) and Factor Loyalty-Authority-Sanctity has 12 indicators (L1 till S4). Each of the indicators is specified to load on only one factor, and measurement errors are not allowed to correlate with other measurement errors.

APPENDIX 6

Figure 3

Competing Model 3: Three correlated factors model. Model is shown for the MFQ-20 and 20-item MFSS

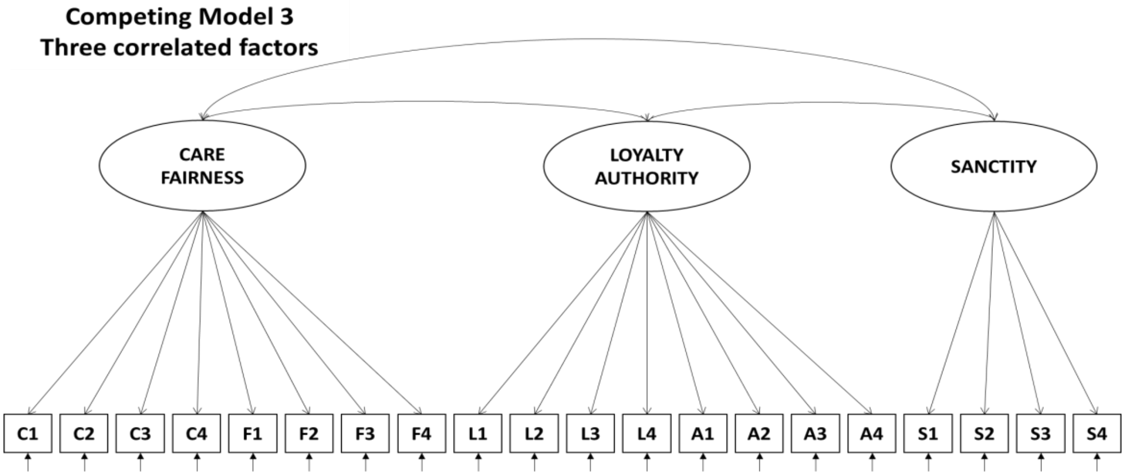

Note: Model 3 represents a three-correlated factor structure in which the three factors are jointly modeled and the relationships between the factors are estimated. Factor Care-Fairness has 8 indicators (C1 till F4), Factor Loyalty-Authority has 8 indicators (L1 till A4) and Sanctity has 4 indicators (S1 till S4). Each of the indicators is specified to load on only one factor, and measurement errors are not allowed to correlate with other measurement errors.

APPENDIX 7

Table 2

Zero order correlations between 20 items of MFQ

	H1	H2	H3	H4	F1	F2	F3	F4	L1	L2	L3	L4	A1	A2	A3	A4	S1	S2	S3	S4
H1	1																			
H2	**.43**	1																		
H3	**.07**	**.07**	1																	
H4	**.19**	**.22**	**.15**	1																
F1	**.20**	**.25**	**.10**	**.13**	1															
F2	**.21**	**.21**	**.14**	**.13**	**.39**	1														
F3	.03	.01	**.06**	**.14**	**.07**	**.06**	1													
F4	**.07**	**.10**	**.15**	**.24**	**.17**	**.08**	**.11**	1												
L1	.01	.04	-.02	**.10**	.04	-.01	.04	-.03	1											
L2	**.07**	**.10**	**.09**	**.06**	**.21**	**.26**	.04	.04	**.27**	1										
L3	-05	**-.06**	-.02	**.05**	**-.12**	-.03	**.07**	-.04	**.25**	**.11**	1									
L4	-.01	.04	-.00	**.13**	-.05	-.02	**.06**	**.06**	**.22**	**.14**	**.23**	1								
A1	.00	**.06**	.03	**.06**	**.12**	**.14**	.03	.01	**.34**	**.28**	**.20**	**.12**	1							
A2	.02	.02	-.04	-.01	**.15**	**.11**	.05	-.04	**.36**	**.25**	**.17**	**.12**	**.32**	1						
A3	**-.10**	**-.06**	**-.06**	-.04	**-.10**	**-.06**	-.01	**-.14**	**.16**	**.11**	**.23**	**.13**	**.12**	**.14**	1					
A4	-.05	-.03	**.10**	.05	**-.06**	.01	**.11**	.04	**.14**	**.12**	**.22**	**.24**	**.36**	**.21**	**.18**	1				
S1	.01	**.06**	.01	.05	**.14**	**.25**	**.06**	-.02	**.19**	**.27**	**.12**	**.12**	**.35**	**.27**	**.09**	**.22**	1			
S2	**.14**	**.13**	**.12**	**.13**	**.25**	**.47**	**.06**	**.06**	**.10**	**.22**	**.05**	-.01	**.16**	**.20**	-.01	.03	**.26**	1		
S3	.03	.05	**.14**	**.13**	**.11**	**.12**	**.13**	**.15**	**.11**	**.13**	**.05**	**.14**	**.15**	**.11**	.03	**.17**	**.17**	**.24**	1	
S4	-.05	-.01	**.07**	**.09**	.01	.01	**.06**	.02	**.21**	**.15**	**.13**	**.17**	**.17**	**.18**	**.17**	**.21**	**.11**	**.13**	**.29**	1

H= Harm ; F= Fairness ; L= Loyalty ; A= Authority ; S= Sanctity

Significant coefficients indicated in bold

Table 3

Zero order correlations between 20 items of MFSS

	ssH1	ssH2	ssH3	ssH4	ssF1	ssF2	ssF3	ssF4	ssL1	ssL2	ssL3	ssL4	ssA1	ssA2	ssA3	ssA4	ssS1	ssS2	ssS3	ssS4
ssH1	1																			
ssH2	**.52**	1																		
ssH3	**.43**	**.35**	1																	
ssH4	**.40**	**.33**	**.47**	1																
ssF1	**.24**	**.23**	**.38**	**.27**	1															
ssF2	**.21**	**.21**	**.32**	**.22**	**.30**	1														
ssF3	**.26**	**.29**	**.36**	**.30**	**.36**	**.23**	1													
ssF4	**.26**	**.30**	**.36**	**.29**	**.26**	**.30**	**.40**	1												
ssL1	**.22**	**.23**	**.38**	**.28**	**.35**	**.33**	**.24**	**.35**	1											
ssL2	**.20**	**.21**	**.27**	**.31**	**.17**	**.17**	**.22**	**.22**	**.27**	1										
ssL3	**.09**	**.09**	**.16**	**.13**	**.14**	**.12**	.04	**.14**	**.32**	**.24**	1									
ssL4	**.17**	**.20**	**.27**	**.23**	**.24**	**.28**	**.20**	**.24**	**.30**	**.30**	**.29**	1								
ssA1	**.22**	**.18**	**.34**	**.28**	**.27**	**.25**	**.15**	**.21**	**.35**	**.33**	**.25**	**.25**	1							
ssA2	**.31**	**.26**	**.46**	**.37**	**.36**	**.35**	**.24**	**.36**	**.41**	**.30**	**.27**	**.32**	**.50**	1						
ssA3	**.22**	**.20**	**.37**	**.30**	**.36**	**.22**	**.28**	**.36**	**.39**	**.21**	**.27**	**.26**	**.31**	**.47**	1					
ssA4	**.23**	**.14**	**.30**	**.23**	**.25**	**.23**	**.12**	**.17**	**.29**	**.19**	**.14**	**.15**	**.39**	**.32**	**.29**	1				
ssS1	**.18**	**.13**	**.24**	**.19**	**.19**	**.20**	**.10**	**.20**	**.30**	**.24**	**.23**	**.18**	**.32**	**.28**	**.27**	**.28**	1			
ssS2	**.17**	**.19**	**.19**	**.25**	**.12**	**.12**	**.18**	**.18**	**.20**	**.29**	**.18**	**.19**	**.18**	**.25**	**.18**	**.10**	**.19**	1		
ssS3	**.20**	**.15**	**.26**	**.20**	**.16**	**.12**	**.01**	**.08**	**.20**	**.22**	**.21**	**.20**	**.24**	**.24**	**.18**	**.20**	**.33**	**.18**	1	
ssS4	**.34**	**.26**	**.37**	**.30**	**.22**	**.21**	**.22**	**.26**	**.29**	**.28**	**.18**	**.21**	**.26**	**.34**	**.33**	**.23**	**.27**	**.36**	**.25**	1

H= Harm ; F= Fairness ; L= Loyalty ; A= Authority ; S= Sanctity

ss= items belonging to Moral Foundations Sacredness Scale -

Significant coefficients indicated in bold

Table 4

Zero order correlations between items of MFQ and MFSS

MFQ → MFSS ↓	H1	H2	H3	H4	F1	F2	F3	F4	L1	L2	L3	L4	A1	A2	A3	A4	S1	S2	S3	S4
ssH1	**.09**	**.07**	**.33**	.05	**.10**	**.11**	.02	**.07**	-.00	.04	-.04	-.01	.01	-.01	**-.08**	.03	.02	**.07**	**.14**	.03
ssH2	**.12**	**.14**	**.22**	**.07**	**.16**	**.13**	.03	.05	-.04	.04	-.04	.01	.04	-.00	**-.08**	.02	.04	**.07**	**.08**	.03
ssH3	**.13**	**.14**	**.12**	.15	**.19**	**.13**	.01	**.12**	.02	.02	**-.06**	.04	.02	.03	**-.13**	**-.08**	.03	**.10**	**.16**	.02
ssH4	**.07**	**.09**	**.07**	**.12**	**.11**	**.12**	.00	**.09**	.03	-.02	-.00	**.06**	**.08**	.02	-.05	.03	.04	**.12**	**.16**	**.07**
ssF1	.04	**.09**	**.09**	**.07**	**.11**	**.08**	.03	**.09**	.04	.01	-.02	.04	.04	**.07**	-.01	.03	**.06**	.04	**.08**	-.01
ssF2	.04	**.06**	**.06**	.04	**.09**	**.09**	.02	**.10**	**.05**	**.10**	.03	**.09**	**.08**	**.06**	-.03	.01	**.09**	**.06**	**.12**	-.02
ssF3	**.10**	**.09**	**.06**	**.07**	**.18**	**.14**	.01	**.15**	**-.10**	-.03	**-.16**	**-.08**	**-.05**	**-.09**	**-.22**	**-.16**	.00	**.06**	**.06**	**-.08**
ssF4	**.09**	**.09**	**.09**	**.08**	**.13**	**.11**	.03	**.10**	.00	.01	-.01	.03	**.06**	.04	**-.11**	.03	**.07**	.03	**.08**	-.03
ssL1	.03	.04	**.05**	**.07**	.05	**.09**	.05	**.05**	**.15**	**.07**	**.17**	**.13**	**.14**	**.11**	.05	**.11**	**.15**	**.10**	**.15**	.05
ssL2	.04	**.06**	**.06**	**.06**	**.09**	**.08**	.04	**.05**	**.09**	**.07**	.04	**.15**	**.10**	**.09**	**-.08**	**.05**	**.08**	**.11**	**.14**	**.08**
ssL3	.02	.02	.01	**.06**	-.01	.04	.01	-.03	**.12**	**.07**	**.22**	**.15**	**.15**	**.16**	**.09**	**.12**	**.10**	**.06**	**.12**	**.11**
ssL4	**.07**	**.06**	.03	**.06**	**.07**	**.10**	.04	.03	**.06**	**.11**	**.05**	.04	**.08**	**.07**	-.02	.03	**.06**	**.05**	**.11**	.04
ssA1	.02	.03	.06	**.07**	**.07**	**.07**	.01	.04	**.07**	**.06**	**.08**	**.17**	**.14**	**.12**	**.07**	**.06**	**.10**	**.07**	**.16**	.05
ssA2	**.07**	**.06**	**.11**	**.11**	**.12**	**.12**	.03	**.06**	**.10**	**.06**	**.06**	**.13**	**.17**	**.09**	-.03	**.09**	**.12**	**.09**	**.17**	.01
ssA3	**.05**	.04	.04	**.06**	**.05**	**.06**	.01	.04	**.09**	.01	**.08**	**.11**	**.13**	**.10**	.00	**.11**	**.09**	.04	**.18**	.05
ssA4	.00	.05	**.07**	**.06**	**.08**	**.05**	.03	.02	**.05**	**.05**	.04	**.12**	**.06**	**.06**	.02	.01	**.05**	**.06**	**.13**	**.06**
ssS1	.01	.04	**.06**	**.08**	**.08**	.01	**.06**	.04	**.16**	**.06**	**.16**	**.17**	**.16**	**.13**	**.10**	**.12**	**.13**	**.08**	**.21**	**.18**
ssS2	**.08**	**.07**	.05	.00	**.09**	**.10**	.01	.03	**.05**	**.05**	.05	.03	**.09**	**.08**	-.02	.04	**.12**	**.11**	**.12**	.02
ssS3	**.10**	**.06**	**.13**	**.10**	**.09**	**.06**	.01	**.08**	**.12**	**.08**	**.10**	**.10**	**.11**	**.12**	**.06**	.03	**.06**	**.14**	**.21**	**.13**
ssS4	**.06**	.04	**.11**	.05	**.06**	**.10**	**.05**	**.08**	**.05**	**.05**	.03	**.06**	**.08**	**.05**	**-.07**	.03	**.09**	**.15**	**.22**	**.08**

H= Harm ; F= Fairness ; L= Loyalty ; A= Authority ; S= Sanctity

ss= items belonging to Moral Foundations Sacredness Scale -

Significant coefficients indicated in bold
